# A cross-sectional survey investigating women’s information sources, behaviour, expectations, knowledge and level of satisfaction on advice received about diet and supplements before and during pregnancy

**DOI:** 10.1186/s12884-018-1834-x

**Published:** 2018-05-25

**Authors:** Gillian Funnell, Kevin Naicker, John Chang, Natasha Hill, Reem Kayyali

**Affiliations:** 1 Faculty of Science, Engineering and Computing, Kingston University, Penrhyn Rd, London, KT1 2EE United Kingdom; 2 Croydon University Hospital, 530 London Road, Croydon, CR7 7YE United Kingdom

**Keywords:** Expectations, Knowledge, Satisfaction, Nutrition, Pregnancy, Mobile application, Folic acid, Vitamin D, Nutrition

## Abstract

**Background:**

The reported long-term effects of poor maternal nutrition and uptake of recommended supplements before and during pregnancy was the impetus behind this study. Our objectives were to investigate and understand women’s expectations, knowledge, behaviour and information sources used regarding the use of nutrition and vitamin supplements before and during pregnancy.

**Methods:**

A cross-sectional survey using a self-administered questionnaire was undertaken. A purposive sampling technique was used. Women attending the antenatal clinic at Croydon University Hospital during 2015 were invited to take part in the study. The data was analysed using descriptive statistics, paired sample T-tests and Chi-squared tests, with the level of significance set at 5% (*p* < 0.05).

**Results:**

A total of 133 pregnant women completed the survey. Analysis of the results showed that women are currently using electronic resources (33%, *n* = 42) rather than healthcare professionals (19%, *n* = 25) as an information source before pregnancy. Women who sourced information through the internet were significantly more likely to take folic acid (*p* = 0.006) and vitamin D (*p* = 0.004) before pregnancy. Women preferred to receive information from the antenatal clinic (62%, *n* = 83), internet (46%, *n* = 61) and from mobile applications (27%, *n* = 36). Although women believed they had sufficient knowledge (60%, *n* = 80) and had received adequate advice (53%, *n* = 70) concerning the correct supplements to take, this was not demonstrated in their behaviour, with only a small number of women (37%, *n* = 49) taking a folic acid supplement before pregnancy. Women mistakenly perceived the timing of supplement advice as correct, with only a small number of women (18%, *n* = 23) considering the advice on supplements as too late.

**Conclusions:**

Despite the small sample size, this study demonstrated that women did not receive timely and/or accurate advice to enable them to take the recommended supplements at the optimal time. Women had the misconception that they understood the correct use of pregnancy supplements. This misunderstanding may be prevented by providing women intending to become pregnant with a structured, approved electronic source of information that improves their supplements uptake.

## Background

Benefits of positive nutritional interventions can be maximised before conception and during the first 12 weeks of pregnancy [1]. Three individual components that influence an individual’s susceptibility to later life disease include genome, lifestyle and early life development in and ex utero [2]. The thrifty phenotype hypothesis states that nutrition in utero plays an important part in the relationship between early growth and later development of the signs of metabolic syndrome [3]. Deficient maternal diet or placental problems can cause stress to the foetus prompting adaptations to increase chance of survival [2–6]. Adaptations include protecting vital organs such as the brain whilst the development of other systems such as the endocrine system may be compromised [6]. Biological organ and tissue changes which are not apparent at birth may give rise to future health problems. Poor nutrition in vitro can optimise metabolic efficiency and storage for an expected birth environment that will also be nutritionally deprived [6]. Adaptations have a more pronounced detrimental effect when in and ex utero environments do not match. For example, there is evidence of high rates of glucose intolerance in low birth weight individuals that become overweight in early life [3] but low rates of diabetes when there is matching chronic malnutrition both in and ex utero [7, 8].

There is increasing evidence linking inadequate nutrition and deficiency of nutrients during pregnancy with later life obesity [9], cardiovascular disease (CVD) [10], type 2 diabetes (T2DM) and atopic diseases [11]. Obese or diabetic mothers have a higher leptin resistance resulting in decreased satiety and overweight offspring or low birth weight offspring whose accelerated post-natal growth is associated with a greater risk for CVD [10–13]. Higher maternal glycemic diets result in higher birth weights and skin thickness in offspring. Maternal glucose levels have a linear relationship with macrosomia and increased adiposity in offspring [9]. Maternal obesity can result in developmental programming due to DNA methylation of sperm to alter gene expression causing a ‘fuel mediated teratogenesis’ resulting in a general increase in obesity and T2DM in successive generations [9, 13]. Low birth weight offspring have a greater chance of future T2DM development [3, 4, 12, 14], whilst higher birth weights showed increase risk of type 1 diabetes (T1DM) [15].

The National Institute of Health and Care Excellence (NICE) guidelines recommend that pregnant women and women planning to conceive should take supplements of folic acid [16]. Not taking folic acid before and during pregnancy increases the risk of offspring which are small for gestational age (SGA) [17]. Folic acid, 400μg, is recommended daily, starting at least 1 month before pregnancy and during the first 3 months [16]. There is concern about patient adherence on following advice on folic acid supplementation [18]. Women are advised to take a 5 mg/day supplement of folic acid if: they have previously had a baby with a neural tube defect; if they or their partner have a neural tube defect or a family history of neural tube defects; or if the woman has diabetes or takes anti-epileptic medication and other named conditions [1].

NICE also recommends a daily Vitamin D 400 IU supplement to mothers that have not taken Vitamin D supplements during pregnancy [19], exclusively breast fed infants or formula-fed infants receiving less than 500 ml daily, infants and children from 6 months to 5 years and to women who are pregnant and breastfeeding [16, 20, 21]. A systematic review of 42 studies revealed that Vitamin D deficiency measured by food intake during pregnancy causes an increased occurrence of childhood asthma and wheeziness [11]. However, a smaller study (*n* = 500) correlated an increase in asthma and atopic eczema with higher vitamin D serum concentrations [22]. Vitamin D deficiency may be implicated with an increased risk of gestational diabetes, pre-eclampsia [23], osteoporosis, diabetes and some cancers [21]. The mother’s level of vitamin D during pregnancy largely determines the offspring’s vitamin D status [24]. Low Vitamin D is defined as having a plasma concentration of below 25 nm/litre (10 ng/ml) [25]. Symptomatic Vitamin D deficiency is common in children under 5 years old with an occurrence of 7.5 per 100,000 children [26]. Children born to mothers with serum 25-(OH)-D concentration < 50 nmol/L during pregnancy exhibit deficits in bone mineral content at 9 years of age [21].

Healthy Start is a UK government scheme that acts as a nutritional safety net for pregnant women that are currently at least 10 weeks pregnant and families or have a child from 6 months to less than 4 years old and on qualifying benefits and tax credits [27, 28]. Studies have found that the uptake of Healthy Start vitamins ranged between 3 and 10% [29], whilst the uptake of Healthy Start food and milk vouchers were reported to be between 72 to 86% [30, 31]. This difference could exist due to availability of milk and food from a wide variety of outlets. Some studies propose that the uptake of Healthy Start vitamins can be increased to 23% for adults and 20% for children if universal free supplementation was supported by a campaign to increase awareness [31].

A systematic review of internet usage of pregnant women seeking pregnancy related information has reported that women use the internet as a source of information at least once a month with fetal development and nutrition in pregnancy being the most frequent search criteria [32]. Women found the information on the internet to be reliable and useful. However, women did not always discuss the information sourced from the internet with their health providers and this could result potentially in unreliable information leading to inaccurate beliefs e.g. “Eating for two” whilst pregnant. Another systematic review reported that motivational interviewing in brief consultations with healthcare professionals can offer a moderate advantage to changing behaviour as compared to using non-human information sources e.g. leaflets [33].

The potential of long term effects caused by poor nutrition, lack of vitamin D and folic acid supplementation, together with the low uptake rate of the Healthy Start vitamin supplements was the impetus behind our investigations. The aim of the study was to investigate women’s expectations, knowledge, behaviour, information sources and identify any gap between expectations and level of satisfaction on advice received about nutrition and vitamin supplementation before and during pregnancy.

## Methods

### Study area, aims and design

This study was conducted in the winter of 2015 at Croydon University Hospital (CUH), antenatal clinic. A purposive sampling strategy was employed to recruit the participants. London has reported the highest birth rates in the UK since 2012 with the Croydon borough reporting the second highest birth rate since 2012 [34]. CUH is the largest hospital in the Croydon area and was chosen due to existing research collaborations between the academic institute and the trust. This study aimed to investigate pregnant women’s expectations, knowledge and behaviour, together with the information sources women utilised to find out about recommended vitamin supplements that should be taken before and during pregnancy. The survey was constructed after a literature search showed a gap in knowledge of information sources used by pregnant women, this together with the NICE antenatal guidelines for supplements and antenatal care [16, 19, 33] informed the design and content of the survey. The questionnaire was in 3 sections: Section 1 sought the medical and pregnancy history of respondents. Section 2 focused on dietary and supplement changes, information sought and checking by healthcare professionals. Women were asked about changes to their intake before and during pregnancy of different foodstuffs, such as staple carbohydrates (eg. bread, rice, pasta), protein (eg. fish, meat, eggs), dairy (cheese, milk etc.), fruit and vegetables and high sugar foods (eg. cakes, sweets, biscuits). A list of common supplements including folic acid, vitamin D, iron, omega oils, Healthy Start Vitamins was provided for women to indicate which, if any, they took before and during pregnancy. Women were asked about the sources from which information about diet and supplements were sought or acquired. Additionally, there were questions concerning the timing of advice received, expectations, satisfaction and understanding of the benefits of taking supplements and good nutrition whilst pregnant. The final section covered patient demographics.

Prior to the study, a pilot study involving 5 local pregnant women was carried out and the survey was deemed to be easy to complete and understand with no amendments needed. The final self-administered questionnaire was offered to all pregnant women attending the clinic during the study period.

Women were asked to verbally consent to participate in the study. The anonymity of all participating women was ensured by not recording any names or other personal identifiers, written consent was therefore not deemed necessary by the ethics committee. A participant information sheet was provided for each woman for information. Based on recommendation from the antenatal team, women were not approached if they looked ill, were upset or were obviously experiencing discomfort, nor if they were in a large group where it was not possible to identify who was pregnant. To estimate the required sample size, the number of deliveries at CUH in 2013–14 was used [35]. This was documented as 2577 deliveries. This translates to approximately 215 antenatal visits within a month. A sample size calculation showed that 139 surveys need to be completed at 95% confidence interval and a +/− 5% margin of error to be representative of the typical population visiting an antenatal unit per month [36]. Therefore, 191 women (*n* = 191) were approached in the waiting area.

### Ethical considerations and data analysis

The Delegated ethics committees for the Faculty of Science, Engineering and Computing at Kingston University approved the study on 22nd December 2014 (ref: 1213/045). Quantitative data from each questionnaire was analysed using Microsoft Excel 2007® and IBM SPSS Statistics Version 23® through descriptive statistics and means compared using a paired sample T-test and Chi-Squared test for independence with the level of significance set at 5% (*p* < 0.05).

## Results

### Socio demographic data

Questionnaires were offered to 191 women (*n* = 191), 33 women declined to answer the questionnaire, 16 women who were approached did not speak English well enough to fill in the questionnaire. Thus, a total of 142 questionnaires were returned. Only 133 of the questionnaires were used as 9 were classified as being too incomplete to use as less than half the questions were answered. Therefore, the response rate was 69.6% (*n* = 133/191). The largest group 38% (*n* = 43) of women classified themselves as white. This included women from Poland**,** Romania, France and Spain. 32% (*n* = 39) had a degree, with 16% (*n* = 20) specifying they had a further degree. 3% (*n* = 4) had no qualifications. Most responders (59%, *n* = 79) were in their 2nd trimester and thus due to attend the clinic at 28 weeks. This was the first pregnancy for 41% (*n* = 54) of women. 77% (*n* = 103) of women had no health issues. The age and ethnic distribution of the sample is within range of the 2011 Croydon census data increasing the validity and reliability of results [37]. The demographic characteristics of the women who provided data are summarised in Table 1.

**Table 1:** Patient socio demographic data

Characteristic	*n* (%)
Age in years (*n* = 115)	
Below 20	5 (4%)
21–25	23 (20%)
26–30	29 (25%)
31–35	39 (34%)
36–40	14 (12%)
Over 41	5 (4%)
Race (*n* = 121)	
White	46 (38%)
Asian/Asian British	31 (26%)
Black, Caribbean, African/Black British	28 (23%)
Mixed/Multiple ethnic groups	8 (7%)
Other ethnic group	4 (3%)
Arab/Persian	2 (2%)
Chinese	2 (2%)
Qualifications (*n* = 123)	
GCSE or equivalent	56 (46%)
Degree e.g. BA/BSc	39 (32%)
A Level	35 (28%)
Further Degree	20 (16%)
Certificate of Higher Education	17 (14%)
HND BTech	5 (4%)
No qualifications	4 (3%)
Other	2 (2%)
Number of Weeks Pregnant (*n* = 133)	
1st Trimester (≤ 12 Weeks)	18 (14%)
2nd Trimester (13–28 Weeks)	79 (59%)
3rd Trimester (≥ 28 Weeks)	36 (27%)
Number of Children (*n* = 133)	
1st Pregnancy	54 (41%)
One child	45 (34%)
Two Children	16 (12%)
Three Children	9 (7%)
> 3 Children	9 (7%)
Current Health Issues (*n* = 133)	
None	103 (77%)
T1DM	3 (2%)
T2DM	0 (0%)
GDM	4 (3%)
Pre-eclampsia	4 (3%)
Other	19 (14%)

### Women’s preferential sources of information

As per Table 2, women more readily sought nutritional information during as compared to before pregnancy. The internet (26%, *n* = 34; 42%, *n* = 56) and the general practitioner (GP) (14%, *n* = 18; 29%, *n* = 39) were the most popular information sources whilst the community pharmacist (0%, *n* = 0; 2%, *n* = 2) and antenatal clinic (5%, *n* = 7; *n* = 29, 22%) and mobile applications (7%, *n* = 9; 17%, *n* = 22) were least popular before and during pregnancy respectively. A paired samples T-test showed that women statistically increased the sourcing of information from GPs (*p* < 0.001), antenatal clinics (*p* < 0.001), the internet (*p* = 0.001) and mobile applications (*p* < 0.001) during pregnancy as compared to before.

**Table 2:** Patient information sources before and during pregnancy

	GP	AC	CP	IT	MA
BP	18	7	0	34	9
BP%	14%	5%	0%	26%	7%
DP	39	29	2	56	22
DP %	29%	22%	2%	42%	17%
p	< 0.001^a^	< 0.001 ^a^	0.158	0.001 ^a^	< 0.001 ^a^

*BP* before pregnancy, *DP* during pregnancy, *GP* General Practitioner, *AC* Antenatal Clinic, *CP* Community Pharmacist, *IT* internet, *MA* mobile applications

^a^Statistically significant increase in using information sources during versus before pregnancy assessed using paired samples T-test

As per Table 3, women received the most verbal advice (44%, *n* = 58) and leaflets (23%, *n* = 31) on vitamin supplementation from the antenatal clinic.

**Table 3:** Location of advice obtained (leaflet or verbal) and vitamin supplementation obtained before and during pregnancy

Location	Vitamins obtained	Verbal advice	Leaflet advice
Antenatal clinic	14 (11%)	58 (44%)	31 (23%)
Nurse @ GP surgery	0 (0%)	35 (26%)	15 (11%)
Pharmacy	63 (47%)	3 (2%)	1 (1%)
Prescription @ pharmacy	25 (19%)		
Supermarket	36 (27%)		
Internet	6 (5%)		

Table 4 shows that women preferred electronic (63%, *n* = 43) rather than healthcare professionals (37%, *n* = 25) as an information source before pregnancy. However, there was little difference between women’s preference of electronic (53%, *n* = 78) versus healthcare professionals (47%, *n* = 70) as an information source during pregnancy, with women having more contact with health care professionals when they are pregnant.

**Table 4:** Healthcare Professionals versus Media as information sources before and during pregnancy

	Before	During
Electronic Information (IT+MA)	43 (63%)	78 (53%)
Healthcare Professionals (GP + AC + CP)	25 (37%)	70 (47%)

*GP* General Practitioner, *AC* Antenatal Clinic, *CP* Community Pharmacist, *IT* Internet, *MA* Mobile Applications

During pregnancy, women showed a difference between their actual as compared to their desired information sources. Table 5 shows that the largest difference in desired versus actual nutritional information during pregnancy in order of preference is from the antenatal clinic (40%, *n* = 54), community pharmacist (19%, *n* = 26), mobile phone applications (10%, *n* = 14) and the internet (4%, *n* = 5).

**Table 5:** Actual versus desired information sources during pregnancy

	AC	CP	MA	IT
AI	29	2	22	56
AI%	22%	2%	17%	42%
DI	83	28	36	61
DI %	62%	21%	27%	46%
DI-AI	54	26	14	5
DI%-AI%	40%	19%	10%	4%

*AC* Antenatal Clinic, *CP* Community Pharmacist, *IT* internet, *MA* mobile applications, *AI* actual information sourced, *DI* desired information source

None of the women had received any nutritional information from their pharmacist before pregnancy (Table 2). However, women (21%, *n* = 28) desired to receive nutritional information from community pharmacists during pregnancy (Table 5) and most women (47%, *n* = 63) obtained their vitamins from a pharmacy. Interestingly, women reported the internet (42%, *n* = 56) as the highest electronic information source used (Table 2). However, the difference between the desired, compared to the actual use of mobile applications (10%, *n* = 14) was statistically greater than the internet (4%, *n* = 5) as an information source (*p* = 0.012) (Table 5).

### The use of vitamin supplements before and during pregnancy

There was a statistically significant difference in usage of folic acid, vitamin D-folic acid combination and commercial pregnancy supplements during than before pregnancy. Table 6 shows that the largest difference in the usage of supplements before as compared to during pregnancy is for commercial supplements for pregnancy (37%, *n* = 68, *p* < 0.001). This may be explained by the observation that at the booking-in appointment women were handed a bag of samples of products and advertising information relevant to pregnant women that included a month’s sample of a commercially branded vitamin supplement and vouchers. On the other hand, only a small number of women reported using the cost-effective Healthy Start supplements before (*n* = 2) and during (*n* = 3) pregnancy. A similar trend was seen for vitamin D consumption. Moreover, only a small number of women took folic acid (26%, *n* = 35) or a vitamin D-folic acid combination (3%, *n* = 4) before becoming pregnant. It can be noted that 41 women reported using more than one supplement during pregnancy. However, over half of women (55%, *n* = 73) took no supplements before they were pregnant, with less than 10% (*n* = 10) reporting taking no supplements during their pregnancy to date.

**Table 6:** The use of supplements before and during pregnancy

	NS	FA	VD	VDFA	HSV	CS
BP	73	35	7	4	2	8
BP%	55%	26%	5%	3%	2%	6%
DP^b^	10	56	13	14	3	79
DP %	8%	42%	10%	11%	2%	59%
p	< 0.001^a^	0.007^a^	0.158	0.004^a^	0.566	< 0.001^a^

*BP* before pregnancy, *DP* during pregnancy, *NS* no supplements taken, *FA* folic acid, *VD* Vitamin D, *VDFA* Vitamin D and folic acid combination, *HSV* Healthy Start Vitamin, *CS* Commercial Branded Pregnancy supplements

^a^Statistically significant increase in the use of supplements during versus before pregnancy assessed using paired samples T-test

^b^Women (*n* = 41) took more than one supplement during their pregnancy

### Dietary changes made before and during pregnancy

As per Table 7, women reported that they increased their consumption of healthier foods e.g. fresh fruit and vegetables (66%, *n* = 88) and reported they decreased their consumption of unhealthy foods e.g. cakes, biscuits, chocolates, or sweets during pregnancy (44%, *n* = 59) as compared to before (14%, *n* = 18). Only 44% (*n* = 50) stated that they were advised not to “eat for two” during pregnancy.

**Table 7:** Dietary changes made before and during pregnancy

	IBP	DBP	IDP	DDP
FFV	31 (23%)	6 (5%)	88 (66%)	8 (6%)
CBCS	14 (11%)	18 (14%)	18 (14%)	59 (44%)

*IBP* increased before pregnancy, *DBP* decreased before pregnancy, *IDP* increased during pregnancy, *DDP* decreased during pregnancy, *FFV* fresh fruits and vegetables, *CBCS* cakes, biscuits, chocolates, and sweets

### Explanation, timing and checking compliance of folic acid, vitamin D and nutrition, expectations, knowledge and level of satisfaction

Women expected to receive advice on nutrition (59%, *n* = 78) and vitamin supplementation (59%, *n* = 79) from the healthcare professionals they met in hospital (Table 8).

**Table 8:** Expectation, satisfaction, and knowledge of dietary and vitamin supplementation advice

	Nutritional	Vitamin
Expectation of advice	78 (59%)	79 (59%)
My knowledge is adequate	92 (69%)	80 (60%)
Satisfaction of advice provided	63 (47%)	70 (53%)

However, women reported not receiving advice to take Vitamin D (35%, *n* = 46), folic acid (16%, *n* = 21) and on improving nutrition (26%, *n* = 35) (Table 9). Furthermore, women did not receive an explanation of the importance of taking folic acid (26%, n = 35) and Vitamin D (50%, *n* = 67). Results showed that checking of compliance at the antenatal clinic for folic acid (59%, *n* = 96) and Vitamin D (33%, *n* = 44) was not consistent. Nevertheless, women believed their knowledge of nutrition (69%, *n* = 92) and vitamin supplementation (60%, *n* = 80) (Table 8) was adequate. Women also thought that they received advice on Vitamin D (38%, *n* = 50), folic acid (59%, *n* = 78) and nutrition (49%, *n* = 65) at the correct time (Table 9) with approximately 50% of women being satisfied with the advice provided on nutrition (47%, *n* = 63) and vitamin supplementation (53%, *n* = 70) (Table 8).

**Table 9:** Timing/Explanation of advice received and compliance checking

	Vitamin D	Folic acid	Nutrition
Timing -Too early	1 (1%)	3 (2%)	4 (3%)
Timing -Just right	50 (38%)	78 (59%)	65(49%)
Timing -Too late	10 (8%)	13 (10%)	7 (5%)
Not advised to use/change	46 (35%)	21 (16%)	35 (26%)
Advised reason for	50 (38%)	86 (65%)	
Not advised reason for	67 (50%)	35 (26%)	
Not sure if advised reason for	7 (5%)	6 (5%)	
Compliance checked	44 (33%)	96 (72%)	
Compliance not checked	66 (50%)	24 (18%)	
Not Sure if compliance checked	8 (6%)	3 (2%)	

### Behaviour: Information sourced versus supplementation before pregnancy and dietary changes before pregnancy

Women who accessed information from the internet before pregnancy statistically significantly increased their consumption of folic acid (*p* = 0.006), Vitamin D (*p* = 0.004) and commercial branded pregnancy supplements (*p* = 0.03) and fresh fruit and vegetables (*p* = 0.017) (Table 10). There was no statistically significant relationship between women sourcing information from GPs or the antenatal clinic and taking supplements before pregnancy. There was also no statistically significant relationship between being previously pregnant and taking any supplements before pregnancy.

**Table 10:** Statistical Significance of Effect of Information Sourced on Supplementation and Dietary Changes Before Pregnancy

	FA	VD/FA	VD	HSV	CS	Increased FFV	Decreased CBCS
GP	0.471	0.426	0.286	0.131	0.200	0.940	0.058
AC	0.311	0.635	0.525	0.739	0.201	0.737	0.953
IT	0.006^a^	0.979	0.004^a^	0.447	0.030^a^	0.017^a^	0.420
MA	0.775	0.143	0.468	0.704	0.237	0.465	0.434
PP	0.477	0.522	0.364	0.787	0.604		

*PP* previously pregnant, *GP* General Practitioner, *AC* Antenatal Clinic, *IT* internet, *MA* mobile applications, *FA* folic acid, *VD/FA* Vitamin D and folic acid, *HSV* Healthy Start Vitamin, *CS* commercial branded pregnancy supplements, *FFV* fresh fruits and vegetables, *CBCS* cakes, biscuits, chocolates, and sweets

^a^Statistically significant changes made when information is sourced before pregnancy assessed using paired samples T-test

## Discussion

This study revealed women’s information sources, behaviour, expectations, knowledge and level of satisfaction concerning advice about good nutrition and recommended supplements before and during pregnancy. Making healthy dietary changes and taking the recommended vitamin supplements especially before conception and during the first 12 weeks of pregnancy has the potential to maximise health benefits for the offspring.

Women looked more for information from electronic sources e.g. the internet than healthcare professionals both before and during pregnancy. This preference for using electronic media as an information source before pregnancy is a shared outcome found in systematic reviews of similar studies [32]. Women who sourced information on the internet showed a statistically significant correlation with taking folic acid (*p* = 0.006), vitamin D (*p* = 0.004) and increasing dietary intake of fresh fruits and vegetables (*p* = 0.017) before pregnancy. However, the internet may provide information not supported by NICE guidelines resulting in mistaken beliefs e.g. “Eating for two” whilst pregnant and taking supplements that are not recommended. During this study (March 2015), the NHS website recommended folic acid but did not recommend Vitamin D, it has since been updated [38]. There is an opportunity for healthcare professionals to direct women to credible electronic information sources. Women were interested in using mobile applications as electronic sources of information (27%, *n* = 36). Validated mobile applications can deliver recommended information in timely meaningful ‘digestible’ pieces and have the potential to remind and update mothers of the latest nutritional guidance and antenatal tests, preventing confusion from conflicting information through an enhanced user experience [39, 40].

During pregnancy women had the opportunity to speak to healthcare professionals e.g. at antenatal clinics, therefore there was an increase in women obtaining information from these sources as well as an increase from electronic sources. Healthcare professionals were the information source women most desired to access during pregnancy. This is a shared outcome with a systematic review of similar studies [33]. Healthcare professionals, with the exception of pharmacists, are not easily accessible to women who are planning pregnancy. The inclusion of a dietitian in the antenatal team has been suggested [41]. A dietitian who routinely engages with all women early on in pregnancy would enable the provision of targeted information concerning nutrition and supplements. However, encouraging healthcare professionals to act as an information source before pregnancy may enable more women to have the opportunity of benefiting from good nutrition and the recommended supplements at an optimal time, rather than waiting for the first antenatal clinic appointment when it is too late to start taking folic acid.

Women expected to receive advice both on nutrition and nutritional supplementation from healthcare professionals. Women also stated that their knowledge of nutrition and nutritional supplements was adequate and they were satisfied with the advice they received on nutrition and supplements. However, the statistically significant difference in the use of folic acid (*p* = 0.007), vitamin D and folic acid combination (*p* = 0.004), and commercial branded pregnancy supplements (*p* < 0.001) shows that women took more of these supplements during pregnancy than before. Although women felt that their knowledge was adequate and were satisfied with the advice received, they did not take folic acid before pregnancy or vitamin D as recommended in the NICE guidelines. In comparison, a study in Ireland found that over 57% of pregnant women did not take folic acid before pregnancy, with 35% not knowing that this was recommended but, 96% took folic acid during early pregnancy [42]. Interestingly, only 11% of women in our study stated that folic acid advice was given too late. This highlights a ‘gap’ between the women’s expectations and the desired clinical outcomes.

Some women were not advised as to the reason to take folic acid and vitamin D. It is important that women are provided with the rationale for taking each supplement. Providing such explanations and involving individuals in healthcare decisions is recommended by NICE to help adherence [43]. Communication with healthcare care professionals before pregnancy could ensure that a consistent message is provided and allow for questions and discussion. The timing and the explanation of nutritional information is important to the health of both mother and baby. Women are not being educated to know what supplements they should take and when they should start them. Provision of timely, trusted and correct advice would help in preventing any confusion which may arise from using different information sources and may reduce queries during midwife and GP appointments. The antenatal clinic ‘booking in’ appointment (at approximately 10 weeks) is too late in pregnancy to start taking folic acid, but this is often women’s first contact with the antenatal clinic. Previous pregnancy did not result in women taking folic acid (*p* = 0.477), Vitamin D and folic acid combination (*p* = 0.522), Healthy Start vitamins (*p* = 0.787) or commercially branded pregnancy vitamins (*p* = 0.604) before their next pregnancy. This indicates that women had not retained the information they should have received in earlier pregnancies.

The high uptake of commercially branded vitamins during pregnancy were encouraged by its free provision at the antenatal booking-in appointment. Women will take supplements if they are provided free under the endorsement of a health care professional. The low uptake of the Healthy Start vitamin scheme is a missed opportunity to provide free vitamins to women.

Women desired to use community pharmacists as an information source, yet during pregnancy, few reported asking a community pharmacist for information. Interestingly, pharmacies (*n* = 63) represented the most frequented location for obtaining vitamins, but the lowest resource accessed for verbal (*n* = 3) and leaflet advice (*n* = 1) by women. Women may not be aware of community pharmacist’s ability to provide information on nutrition and supplements for pregnancy [44]. Community pharmacists are an accessible source of information and need to proactively advertise their nutritional competency, especially in relation to promoting the importance of healthy nutrition and supplements before pregnancy. Community pharmacies should promote the uptake of cost-effective pregnancy vitamins e.g. Healthy Start vitamins [45]. Community pharmacists have great potential to be an excellent information source for women who are planning to become, or who are pregnant and should encourage women to enroll on the Healthy Start scheme if they are eligible. Community pharmacists could help in ensuring that information given to women is in accordance with current guidelines. e.g. starting folic acid at least 1 month before pregnancy and continuing for 3 months after and discussing the role of folic acid to prevent neural tubular defects and SGA.

Healthcare professionals did not always check women’s adherence to taking supplements with 20% of women not being asked if they were continuing to take folic acid and 56% not asked if they were taking vitamin D. Healthcare professionals need to question women to confirm supplementation and this should be part of their checklist.

Even though electronic sources are popular information resources for pregnant women, existing internet sites and mobile application were unable to adequately educate and motivate women to take the required supplements before pregnancy nor inform them of Healthy Start Vitamins. Women have reported a desire to talk to healthcare professionals e.g. antenatal clinic nurses and community pharmacists. This is a shared outcome with an Australian study, which reported that advice from healthcare professionals was regarded as the most reliable by women [33]. Systematic reviews state that motivational interviewing by such healthcare professionals can offer a moderate advantage in helping change behavior when compared with information only sources e.g. internet and mobile applications [33]. Women also statistically significantly desired mobile applications as the most preferred electronic source of information (*p* = 0.012). A mobile application for childbearing age women that allows direct contact with healthcare professionals may be a way forward to encourage women to eat healthily and take folic acid before conception and during pregnancy. The researchers have not found mobile applications that offer this feature to support pregnant women in the UK during the study period.

### Limitations

The study was conducted in the period from February 23rd to March 5th 2015 and is reflective of this environment at that point in time. This study focused on pregnant women knowledge and behaviour with regards to supplements, therefore the important issues involved in pregnancy for overweight women or overeating in pregnancy were not examined in this study. The sample size is small and constituted only of women who attended the antenatal clinic at CUH and thus results may not be representative of other locations. Furthermore, the questionnaire for this study is in English and therefore only women who had an adequate understanding of English could participate. This also limited the sample size. Women were not asked if their pregnancy was planned or unplanned as that was not deemed appropriate by the ethics committee. However, the response to this question would have revealed if women who planned their pregnancy took the recommended nutritional supplements and changed to a healthier diet. It would have been interesting to investigate the specific websites that women had visited before pregnancy as this would have enabled an assessment of the content of those websites that influenced the uptake of supplements before pregnancy. However, this may be problematic as websites can change content rapidly and it would be difficult to confirm the specific version that had been browsed.

## Conclusions

This study found that women who used electronic information sources before pregnancy, were more likely to take folic acid at the correct time, but this uptake was still low. In fact, women who are currently using the internet, have expressed a desire to use mobile applications as an information source. An NHS pregnancy mobile application/website that allows direct interaction with preferred healthcare professionals e.g. nurses and community pharmacists could create a platform for motivational interviewing and support women in changing their behaviour to improve healthy nutrition and to use the correct supplements on time before becoming pregnant. Additionally, the endorsement of more government approved commercial applications and websites offering validated pregnancy information would reach more women.

This study highlights the discrepancy between pregnant women’s perceived and actual knowledge: Women thought their knowledge about nutrition and supplements was adequate and were satisfied with the advice they received. However, women did not take nutritional supplements on time exposing women’s lack of knowledge of the recommended guidelines and hence their lack of accurate knowledge. There is currently a gap in the provision of this information at the correct time, that is, before conception to ensure offspring are healthy and to help reduce the future disease burden. All healthcare professionals, including underutilised community pharmacists, in contact with women who are pregnant or thinking of becoming pregnant should be encouraged to promote the importance of taking supplements and good nutrition. Although this may require further relevant training.

## Abbreviations

CUH: Croydon University Hospital
CVD: Cardio Vascular Disease
GP: General Practitioner
NHS: National Health Service
NICE: National Institute of health Care Excellence
SGA: Small for Gestational Age
T1DM: Type 1 Diabetes Mellitus
T2DM: Type 2 Diabetes Mellitus
